# Psychosocial Impact of Brain Tumors: A Cross-Sectional Study on Existential Anxiety in Saudi Arabian Patients

**DOI:** 10.7759/cureus.66082

**Published:** 2024-08-03

**Authors:** Najim Z Alshahrani, Mohamed Baklola, Mohannad A Alzain, Mohamed Terra

**Affiliations:** 1 Department of Family and Community Medicine, Faculty of Medicine, University of Jeddah, Jeddah, SAU; 2 Department of Public Health, Mansoura University, Mansoura, EGY; 3 Faculty of Medicine, King Abdulaziz University, Jeddah, SAU; 4 Faculty of Medicine, Mansoura University, Mansoura, EGY

**Keywords:** resilience, spiritual well-being, quality of life, saudi arabia, brain tumors, death anxiety, existential anxiety

## Abstract

Background

Existential anxiety is a significant concern for patients with life-threatening illnesses like brain tumors. This study explores the prevalence and impact of existential anxiety among brain tumor patients in Saudi Arabia, examining relationships between demographic, clinical, and psychological variables and death anxiety.

Methods

A cross-sectional study was conducted with 120 brain tumor patients from inpatient and outpatient settings at King Abdulaziz University Hospital, King Fahad Hospital, and King Abdullah Medical Complex in Saudi Arabia. Data were collected using the Death Anxiety Scale (DAS), Spiritual Well-Being Scale (SWBS), Meaning in Life Questionnaire (MLQ), and the 12-item Short Form Survey (SF-12). Descriptive and inferential statistics analyzed the relationships between variables.

Results

Females exhibited significantly higher DAS scores (77.9 ± 14.2) compared to males (48.5 ± 19.4) (p < 0.001). Educational attainment was inversely related to DAS, with illiterate patients scoring highest (83 ± 13.5) and those with higher education scoring lowest (47.3 ± 18.2) (p < 0.001). Widowed patients had higher anxiety (68.5 ± 22.1) compared to married (51.4 ± 21.5) and single patients (50 ± 12) (p < 0.001). Monthly income showed an inverse relationship with DAS. Patients with chronic medical conditions reported lower DAS scores compared to those without (p = 0.004). The tumor stage significantly influenced DAS, with third-stage patients showing lower anxiety than those in the first and second stages (p < 0.001). Longer duration since diagnosis was associated with lower DAS scores (p = 0.03).

Conclusion

This study highlights the significant psychosocial impact of brain tumors on Saudi Arabian patients, emphasizing the need to address demographic factors in managing death anxiety. Findings indicate that chronic medical conditions and advanced tumor stages might be associated with lower anxiety, revealing potential resilience factors. The positive influence of spiritual well-being and meaning in life on quality of life underscores the importance of holistic care approaches. Integrating psychological and spiritual support tailored to individual patient demographics could enhance management strategies and improve patient outcomes. Future research should explore longitudinal changes in existential anxiety, the role of cultural factors, and the effectiveness of holistic interventions in reducing anxiety and improving quality of life.

## Introduction

Death is an unavoidable aspect of human existence, yet its uncertain nature can be a significant source of anxiety [1]. Patients diagnosed with incurable diseases confront the reality of death more directly [2]. A diagnosis of a terminal illness, such as cancer, often leads to heightened anxiety and stress [3]. The World Health Organization reported that cancer was the second leading cause of death globally in 2018, and it was projected that by 2020, approximately 11.4 million people would be affected. It is estimated that one in three individuals will develop cancer at some point in their lifetime [4,5].

Advanced cancer is a heterogeneous group of diseases that can increase mortality salience and provoke existential anxiety - a profound anxiety about life's meaning, the inevitability of death, and the existential reality [6]. Patients with primary brain tumors (PBTs) may be at heightened risk of existential anxiety due to the certainty of tumor progression, lack of curative treatments, and poor prognosis of their disease. According to the Saudi Cancer Registry, the incidence of brain cancers among Saudi Arabian residents is relatively low. Brain tumors rank among the top 10 most prevalent cancers in the Gulf Cooperation Council (GCC) countries. The average age-standardized prevalence (ASR) for brain cancer is estimated at 2.4 per 100,000 males and 1.6 per 100,000 females. Saudi Arabia, the largest country in the Middle East with an approximate population of 22 million, reported over 300 new cases of brain cancer in 2016, accounting for 2.8% of all cancer patients, according to the latest Saudi Cancer Registry study [7]. Sustaining “double awareness” and managing existential anxiety may, therefore, be more challenging for PBT patients than for those with other cancers [8].

Existential anxiety is a multidimensional construct made up of psychological and emotional factors that affect one’s daily functioning [9]. Previously established dimensions of existential anxiety include death anxiety, death depression, and death obsession [10]. Death anxiety, which refers to the fear of death and dying, has been endorsed by approximately one-third of individuals with advanced cancer, although rates vary across cancer types [10]. Overall, individuals with elevated death anxiety report more general symptoms of anxiety and depression.

Patients diagnosed with cancer who do not receive adequate psychological support and resources are at heightened risk, as the treatment process and subsequent follow-ups can pose significant threats to their mental and physical well-being [11]. The disease can lead to various negative outcomes, including stress and anxiety, depression, feelings of social isolation, frustration, and fear of death [12]. Morphological studies have explored perceptions of death across three primary dimensions: death depression, death anxiety, and death obsession [13].

Being diagnosed with and living through a life-threatening illness such as cancer is an extremely stressful experience that can significantly impact various facets of an individual's life [13]. Cancer represents a major public health issue both in the United States and globally [14]. Currently, it stands as the second leading cause of death in the United States and is anticipated to overtake heart disease as the leading cause in the coming years [14]. The World Health Organization emphasizes the importance of integrating the spiritual and religious aspects of patients' lives into their overall care [15]. However, evaluating patients' spiritual needs poses challenges, partly due to the complex and sometimes ambiguous nature of spirituality [16]. This complexity is especially evident when distinguishing between religion and spirituality, particularly for individuals who do not identify as religious. Given the diverse range of belief systems and religious practices, defining spiritual needs can be difficult [17]. However, having a clear definition is crucial for achieving a shared conceptual understanding. Spiritual needs relate to the "spirit" component of the human experience and are defined as the needs and desires that individuals have to find meaning, purpose, and value in their lives [18]. These needs can be explicitly religious, but even those who do not adhere to any religious faith or are not part of an organized religion have belief systems that provide their lives with meaning and purpose.

Hampton et al. noted that the spiritual needs, distress, and well-being of patients with terminal illnesses can significantly influence their quality of life (QoL) [18]. Similarly, Arrey et al. suggested that spiritual needs associated with patients' illnesses can impact their mental health, and failing to address these needs may adversely affect their QoL [15]. In cancer care, QoL has become a vital concept, encompassing an individual's subjective evaluation of how an illness or its treatment affects their physical, psychological, social, and overall well-being. It has been demonstrated that assessing QoL in cancer patients can lead to better treatment outcomes and serve as an important prognostic indicator [19]. Numerous studies have explored the connections between spirituality, religious beliefs, and QoL in cancer patients [20,21].

In Saudi Arabia, the increasing incidence of brain tumors has prompted significant advancements in medical treatments and an improvement in survival rates [22]. However, the psychosocial implications, particularly existential anxiety, are less understood in this region. Given the complex nature of brain tumors and their treatment, the impact on patients' mental health, including the development of existential anxiety, merits further exploration [20].

The primary aim of this study is to explore and understand the prevalence and impact of existential anxiety among brain tumor patients in Saudi Arabia. By utilizing a comprehensive set of validated tools, this study seeks to provide a nuanced understanding of how existential anxiety manifests in this specific cultural context and its consequent effects on patients' QoL and psychological health. This study will not only contribute to the global understanding of psychosocial oncology but also provide culturally relevant data that can inform better clinical practices and support systems for patients within the Saudi Arabian healthcare context.

## Materials and methods

Study design and study period

This study employed a cross-sectional design with an analytic component to evaluate the psychosocial impact of brain tumors, particularly focusing on death anxiety among patients in Saudi Arabia. Data collection took place from September 2023 to June 2024.

The study population comprised patients diagnosed with brain tumors. The inclusion criteria required patients to be over 18 years of age and able to provide informed consent. The exclusion criteria included elderly patients with advanced dementia.

Sample size calculations

The study's sample size was determined using MedCalc version 15.8 (MedCalc Software Ltd, Ostend, Belgium), considering the primary outcome of interest, which is the mean score on the Death Anxiety Scale (DAS). A previous study in Saudi Arabia [23] found a mean DAS score of 53.2 with a standard deviation (SD) of 22.4. Based on these parameters, with an alpha error of 0.05 and a study power of 80%, the required sample size was calculated to be 119 participants. This calculation ensures adequate power to detect statistically significant differences in the primary outcome measures.

Study tools

To collect data, five primary sections were employed. First, a structured questionnaire gathered detailed sociodemographic information, including age, gender, marital status, education level, and other relevant variables, providing a comprehensive background on the participants' social and economic contexts.

The DAS was utilized to measure the level of anxiety patients experience concerning death. It includes a series of statements rated on a Likert scale, capturing the intensity of death-related anxiety, thus offering a quantitative measure of this specific psychological construct. The Arabic version of the DAS demonstrated good reliability, with a Cronbach's alpha of 0.90 [24].

The Spiritual Well-Being Scale (SWBS) assessed the spiritual health of the patients, consisting of two subscales: religious well-being (RWB) and existential well-being (EWB). The RWB subscale evaluates the patients' sense of peace derived from their faith, while the EWB subscale assesses their overall sense of life purpose and meaning beyond religious beliefs [25]. The SWBS overall showed good reliability, with a Cronbach's alpha of 0.83. The RWB subscale had excellent reliability with a Cronbach's alpha of 0.90, and the EWB subscale exhibited acceptable reliability with a Cronbach's alpha of 0.75 [25].

The Meaning in Life Questionnaire (MLQ) was used to evaluate the patients' perceived meaning and purpose in life. This instrument consists of two subscales: presence of meaning, which measures the extent to which individuals feel their lives have meaning, and search for meaning, which assesses their drive and efforts to find meaning in life [26].

Finally, the QoL questionnaire, the 12-item Short Form Survey (SF-12), was employed to measure the overall quality of life of the patients. The SF-12 includes items covering physical and mental health domains, providing a comprehensive assessment of participants' well-being. The SF-12 demonstrated acceptable reliability, with the Mental Component Summary (MCS-12) having a Cronbach's alpha of 0.707, and the Physical Component Summary (PCS-12) showing slightly higher reliability with a Cronbach's alpha of 0.743 [27].

Sampling and data collection approach

Convenience sampling was used to recruit participants from inpatient and outpatient settings at King Abdulaziz University Hospital, King Fahad Hospital, and King Abdullah Medical Complex in Saudi Arabia. After obtaining informed consent, participants completed the sociodemographic questionnaire and the four psychological scales (DAS, SWBS, MLQ, and SF-12). Data were collected through face-to-face interviews conducted by trained data collectors, who ensured that the participants understood each item on the questionnaires. The data collectors underwent extensive training before data collection to manage the data's sensitive nature and maintain consistency and accuracy in data gathering.

Statistical analysis

Descriptive statistics were used to summarize the sociodemographic characteristics of the participants. Mean and standard deviation (SD) were calculated for continuous variables, while frequencies and percentages were used for categorical variables. Scores from the DAS, SWBS, MLQ, and SF-12 were calculated according to their respective manuals. For inferential statistics, Pearson correlation tests were conducted to examine the relationships between sociodemographic variables, spiritual well-being, meaning in life, quality of life, and levels of death anxiety. T-tests and ANOVA were used to compare mean scores across different groups based on sociodemographic and clinical characteristics. Python and R software (R Foundation for Statistical Computing, Vienna, Austria) were utilized to create visual representations of the data, including bubble charts and correlation matrices. All statistical analyses were conducted using SPSS software version 26 (IBM Corp., Armonk, NY), with a significance level set at p < 0.05.

## Results

Association between demographic and clinical characteristics with death anxiety scores

A total of 120 participants were included in this study, consisting of 22 females and 98 males. Females exhibited significantly higher DAS scores (77.9 ± 14.2) compared to males (48.5 ± 19.4) (p < 0.001). Educational attainment was inversely related to DAS; illiterate patients had the highest scores (83 ± 13.5), while those with higher education had the lowest (47.3 ± 18.2) (p < 0.001). Marital status was also significantly associated with DAS, with widowed patients showing higher anxiety (68.5 ± 22.1) compared to married (51.4 ± 21.5) and single patients (50 ± 12) (p < 0.001). Monthly income showed an inverse relationship with DAS; patients earning more than 10,000 riyals had the lowest scores (41.1 ± 15.2), whereas those earning 3,000-5,000 riyals had the highest (72.7 ± 17.9) (p < 0.001). Additionally, patients with chronic medical conditions reported lower DAS scores (50.3 ± 21.9) compared to those without (62.8 ± 18.5) (p = 0.004). The tumor stage significantly influenced DAS scores, with third-stage patients showing lower anxiety (30.8 ± 8.2) compared to those in the first (61.5 ± 25.2) and second stages (66 ± 20.2) (p < 0.001). The duration since diagnosis was also a significant factor, with patients diagnosed more than three years ago having a lower DAS (43.1 ± 15.1) compared to those diagnosed less than six months ago (58.2 ± 23.8) (p = 0.03). Overall, the mean DAS for the cohort was 53.8 ± 21.7 (Table 1).

**Table 1:** Demographic and clinical characteristics and their association with the Death Anxiety Scale (DAS) scores in patients with brain tumors. DAS: Death Anxiety Scale; SD: standard deviation; P-value: the probability value indicating the significance of the results.

Variable		Count (%)	Death Anxiety Scale	P-value
			Mean (SD)	
Gender	Male	98 (81.7%)	48.5 (19.4)	<0.001
	Female	22 (18.3%)	77.9 (14.2)	
Educational level	Illiterate	9 (7.5%)	83 (13.5)	<0.001
	Primary education	12 (10%)	63.6 (19)	
	Secondary education	31 (25.8%)	56.2 (23.6)	
	High education	68 (56.7%)	47.3 (18.2)	
Marital status	Single	7 (5.8%)	50 (12)	<0.001
	Married	95 (79.2%)	51.4 (21.5)	
	Widowed	18 (15%)	68.5 (22.1)	
Monthly income (Saudi riyals)	Less than 3,000	22 (18.3%)	60.6 (11.1)	<0.001
	3,000 – 5,000	20 (16.7%)	72.7 (17.9)	
	5,000 – 10,000	23 (19.2%)	61.6 (27.5)	
	More than 10,000	55 (45.8%)	41.1 (15.2)	
Having chronic medical conditions	Yes	86 (71.7%)	50.3 (21.9)	0.004
	No	34 (28.3%)	62.8 (18.5)	
Type of brain tumor	Primary tumor	81 (67.5%)	53.4 (21.5)	0.7
	Secondary tumor	39 (32.5%)	54.8 (22.5)	
Tumor stage	First stage	27 (22.5%)	61.5 (25.2)	<0.001
	Second stage	21 (17.5%)	66 (20.2)	
	Third stage	15 (12.5%)	30.8 (8.2)	
	Unknown stage	57 (47.5%)	51.8 (17.8)	
Duration since diagnosis	Less than 6 months	45 (37.5%)	58.2 (23.8)	0.03
	6 months – 1 year	34 (28.3%)	51.3 (22.1)	
	1 year – 3 years	21 (17.5%)	58.8 (18.5)	
	More than 3 years	20 (16.7%)	43.1 (15.1)	

Prevalence of comorbid chronic diseases among brain tumor patients

**Figure 1:**
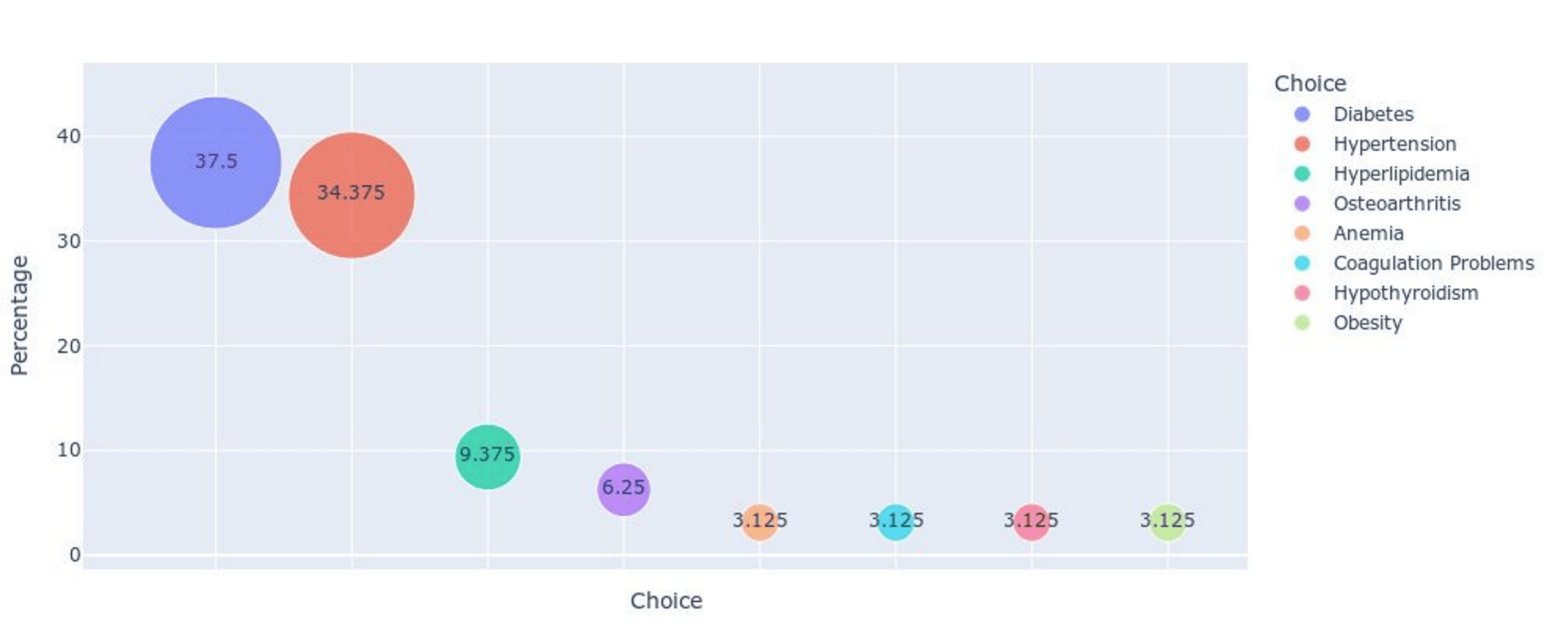
Bubble chart representing the distribution of chronic diseases among patients with brain tumors.

Figure 1 illustrates the distribution of chronic diseases among patients with brain tumors, depicted in a bubble chart format. The most prevalent chronic condition is diabetes, affecting 37.5% of the patients, followed closely by hypertension at 34.37%. Other notable conditions include hyperlipidemia (9.37%) and osteoarthritis (6.25%). Less common chronic diseases among this patient population are anemia, coagulation problems, hypothyroidism, and obesity, each accounting for 3.12% of the cases. The size of the bubbles corresponds to the percentage of patients affected by each condition, highlighting the significant burden of diabetes and hypertension within this group. This distribution underscores the need for comprehensive management strategies that address not only brain tumors but also these prevalent comorbidities to improve overall patient outcomes.

Relationships between demographic variables, spiritual well-being, and the meaning of life

The relationship between demographic variables, spiritual well-being, and the meaning of life was examined, revealing several significant associations (Table 2). Gender differences were notable in the meaning of life scores, with females scoring higher (46) compared to males (41) (p < 0.01), although no significant difference was found in spiritual well-being scores between genders (p = 0.68). Educational level significantly influenced both spiritual well-being (p < 0.01) and the meaning of life (p < 0.01), with illiterate patients having the highest spiritual well-being scores (66) and the lowest meaning of life scores (35). Marital status also played a significant role, as single patients had significantly lower scores in both spiritual well-being (46) and meaning of life (28) compared to married and widowed patients (p < 0.01 for both).

**Table 2:** Relationship between demographic variables, spiritual well-being, and the meaning of life in patients with brain tumors. SD: standard deviation; P-value: the probability value indicating the significance of the results. * Statistically significant p-value < 0.05.

Variable		Spiritual well-being, Mean (SD)	P-value	Meaning of life, Mean (SD)	P-value
Gender	Male	46 (14)	0.68	41 (5)	<0.01*
	Female	46 (12)		46 (5)	
Educational level	Illiterate	66 (10)	<0.01*	35 (1)	<0.01*
	Primary education	46 (11)		45 (3)	
	Secondary education	48 (9)		45 (2)	
	High education	42 (14)		41 (6)	
Marital status	Single	46 (1)	<0.01*	28 (1)	<0.01*
	Married	44 (15)		43 (4)	
	Widowed	55 (5)		43 (5)	
Monthly income	Less than 3,000	49 (6)	<0.01*	43 (5)	<0.01*
	3,000 – 5,000	57 (11)		37 (8)	
	5,000 – 10,000	42 (12)		44 (6)	
	More than 10,000	42 (16)		42 (2)	
Having chronic medical conditions	Yes	44 (14)	0.16	42 (4)	0.6
	No	52 (12)		42 (8)	
Type of brain tumor	Primary tumor	46 (13)	0.44	44 (4)	<0.01*
	Secondary tumor	47 (16)		38 (6)	
Tumor stage	First stage	51 (8)	<0.01*	42 (4)	0.3
	Second stage	54 (18)		40 (5)	
	Third stage	37 (9)		42 (2)	
	Unknown stage	43 (14)		42 (6)	
Duration since diagnosis	Less than 6 months	44 (10)	0.44	43 (5)	<0.01*
	6 months – 1 year	45 (17)		39 (7)	
	1 year – 3 years	48 (6)		44 (3)	
	More than 3 years	49 (20)		41 (1)	

Quality of life scores and their correlates

QoL scores were significantly associated with various demographic and clinical characteristics. Female patients reported higher QoL scores (218.6) than male patients (192.7) (p < 0.01). Educational level showed significant differences in QoL scores (p < 0.01), with illiterate patients scoring the lowest (155) and those with secondary education scoring the highest (218.4). Marital status impacted QoL, with widowed patients having the highest scores (233.3), followed by single (205) and married patients (190.1) (p < 0.01). Additionally, patients without chronic medical conditions had significantly higher QoL scores (233.7) compared to those with chronic conditions (183.2) (p < 0.01). The tumor stage significantly affected QoL, with first-stage patients reporting the highest scores (219.8) and second-stage patients the lowest (171.2) (p < 0.01). Duration since diagnosis did not show a significant impact on QoL scores (p = 0.7) (Table 3).

**Table 3:** Quality of life scores and their association with demographic and clinical characteristics in patients with brain tumors. SD: standard deviation; P-value: the probability value indicating the significance of the results. * Statistically significant p-value < 0.05.

Variable		Quality of life, Mean (SD)	P-value
Gender	Male	192.7 (29.5)	<0.01*
	Female	218.6 (26.3)	
Educational level	Illiterate	155 (18.8)	<0.01*
	Primary education	178.8 (7.7)	
	Secondary education	218.4 (34.2)	
	High education	196.9 (23.9)	
Marital status	Single	205 (2)	<0.01*
	Married	190.1 (27.5)	
	Widowed	233.3 (25.5)	
Monthly income	Less than 3,000	218 (34.5)	<0.01*
	3,000 – 5,000	174.9 (25.8)	
	5,000 – 10,000	205.8 (30.9)	
	More than 10,000	194 (23.8)	
Having chronic medical conditions	Yes	183.2 (21.7)	<0.01*
	No	233.7 (16.2)	
Type of brain tumor	Primary tumor	199.4 (27.1)	0.3
	Secondary tumor	193.6 (36.8)	
Tumor stage	First stage	219.8 (35.9)	<0.01*
	Second stage	171.2 (18.7)	
	Third stage	179.5 (13.7)	
	Unknown stage	201.3 (24.7)	
Duration since diagnosis	Less than 6 months	196.7 (25.7)	0.7
	6 months – 1 year	198.7 (41.9)	
	1 year – 3 years	192 (21.4)	
	More than 3 years	203 (26.6)	

Correlations among psychological factors and quality of life components

**Figure 2:**
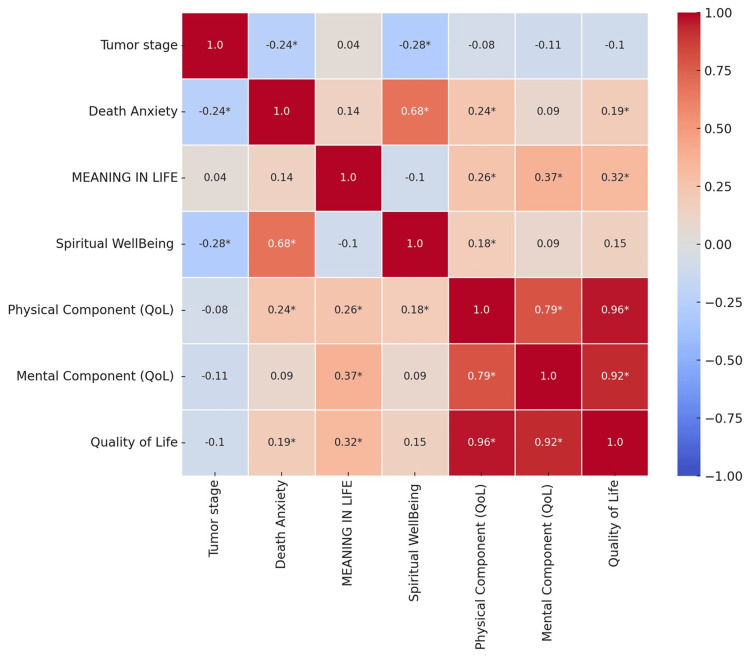
Correlation matrix of psychological and quality of life factors in patients with brain tumors. The X-axis (choice) represents different chronic diseases, and the Y-axis (percentage) represents the percentage of patients with each chronic disease. Bubble size corresponds to the proportion of patients with the respective condition. * Statistically significant p-value < 0.05. QoL: quality of life.

Figure 2 presents the correlation matrix depicting relationships among psychological factors and QoL components in patients with brain tumors. The matrix reveals a significant negative correlation between tumor stage and spiritual well-being (r = -0.28, p < 0.05), indicating that patients in more advanced stages of their tumors tend to have lower spiritual well-being. Death anxiety shows a strong positive correlation with spiritual well-being (r = 0.68, p < 0.05), suggesting that higher levels of death anxiety are associated with higher spiritual well-being. Meaning in life is positively correlated with both the physical (r = 0.26, p < 0.05) and mental components (r = 0.37, p < 0.05) of QoL, as well as overall QoL (r = 0.32, p < 0.05), highlighting the role of perceived meaning in enhancing patients' QoL. The physical component of QoL is highly correlated with the mental component (r = 0.79, p < 0.05) and overall QoL (r = 0.96, p < 0.05), demonstrating that physical health significantly impacts overall QoL. This comprehensive analysis underscores the interconnectedness of psychological well-being and QoL in brain tumor patients, emphasizing the importance of holistic care approaches.

## Discussion

The findings of our study provide important insights into the psychosocial impact of brain tumors on patients in Saudi Arabia. Notably, we observed significant gender differences in DAS scores, with females exhibiting higher levels of anxiety compared to males. Additionally, educational attainment was inversely related to DAS, suggesting that higher education levels may equip individuals with better coping mechanisms and access to information, thereby reducing anxiety. Marital status also played a critical role, with widowed patients showing the highest levels of death anxiety, highlighting the importance of social support systems in mitigating existential fears.

Our findings reveal that females exhibit significantly higher DAS scores compared to males, which is consistent with existing literature in the region that highlights similar trends among female cancer patients [23]. This gender disparity may be influenced by socio-cultural norms in Saudi Arabia, where traditional gender roles often impose greater emotional and caregiving responsibilities on women. Such societal expectations may exacerbate existential anxiety, particularly in the context of life-threatening illnesses [28].

The inverse relationship between educational attainment and DAS suggests that individuals with higher education levels may possess better coping mechanisms and greater access to informational resources, thereby mitigating anxiety. This finding aligns with theories that link educational attainment to improved health literacy and psychological resilience [29]. Moreover, the marital status of patients emerged as a significant factor, with widowed individuals reporting the highest levels of death anxiety. This underscores the critical role of social support systems in buffering against existential fears, particularly in the absence of a spouse [23,30]. Being married or having a close support network can provide emotional and practical support, which helps mitigate existential fears and anxiety [31]. Widowed patients, on the other hand, might experience heightened anxiety due to the loss of their primary source of emotional support, contributing to their higher DAS.

Our study's findings are further supported by a meta-analysis on death anxiety in cancer patients, which consistently found higher levels of anxiety among females compared to males [30]. This suggests that the observed gender differences in our study may be part of a broader, culturally influenced phenomenon. Understanding the unique socio-cultural context of Saudi Arabia, including gender-specific caregiving responsibilities and societal expectations, is essential for interpreting these differences. Additionally, the limited access to mental health resources for women in the region may contribute to heightened levels of anxiety.

An intriguing and somewhat counterintuitive finding in our study was that patients with chronic medical conditions reported lower DAS scores compared to those without such conditions. This observation contrasts with findings from previous research on death-related distress in adult PBT patients, where generalized anxiety was identified as a significant predictor of death anxiety [32]. One plausible explanation for this discrepancy is that patients with chronic conditions may develop psychological resilience over time. The continuous management of chronic illnesses might equip these patients with coping strategies and a level of psychological adaptation that mitigates the anxiety typically associated with a new, life-threatening diagnosis such as a brain tumor [33].

Furthermore, familiarity with the healthcare system and frequent interactions with healthcare professionals could contribute to a sense of preparedness and reduce uncertainty, thereby lessening death anxiety. These patients may perceive their brain tumor as another health challenge to be managed, rather than an entirely new and overwhelming threat. This perspective could diminish the incremental anxiety that typically accompanies such diagnoses [33].

Another notable finding was the significantly lower DAS scores in third-stage tumor patients compared to those in the first and second stages. This finding contrasts with the common assumption that advanced disease stages are associated with higher anxiety levels. However, it is supported by the cluster analysis of death-related distress in PBT patients, which found that patients further out from their diagnosis tend to be in the resilience cluster [32]. This phenomenon might be due to psychological adaptation over time and the development of coping strategies or acceptance regarding their condition [34]. Patients who have lived longer with their diagnosis might have adjusted to their situation, resulting in lower death anxiety [34].

QoL scores in our study were higher among females and patients without chronic medical conditions [35,36]. This is consistent with findings from the Preston Robert Tisch Brain Tumor Center, which reported that greater spiritual well-being is associated with better health-related quality of life (HRQoL) [36]. The positive correlation between spiritual well-being and QoL highlights the importance of addressing spiritual needs in clinical care [36]. Patients with higher spiritual well-being may have a stronger sense of purpose and meaning in life, which can improve their overall well-being and QoL [37]. The role of meaning in life in enhancing QoL was also evident in our study, with significant correlations between the meaning of life scores and QoL components [38]. This finding aligns with the Turkish study on meaning in life, which demonstrated that a higher sense of meaning in life significantly impacts various aspects of QoL in cancer patients [38]. Patients who perceive a greater sense of meaning in their lives tend to report better overall well-being, underscoring the importance of addressing existential concerns in clinical care [37,38].

Future research should focus on longitudinal studies to better understand the progression of death anxiety over time in patients with brain tumors. Such studies could investigate how death anxiety evolves from diagnosis through various stages of treatment and disease progression. Additionally, exploring the potential moderating effects of chronic medical conditions, psychological resilience, and adaptation on death anxiety would provide valuable insights. These studies should also consider the impact of continuous healthcare engagement and familiarity with medical environments on patients' anxiety levels. Understanding these dynamics can inform the development of targeted interventions aimed at reducing death anxiety and improving the overall QoL for brain tumor patients.

Limitations

Our study has several limitations that should be acknowledged. First, the cross-sectional design precludes any causal inferences regarding the relationships between demographic, clinical, and psychological factors and death anxiety. Second, the sample size, while adequate, limits the generalizability of the findings to a broader population of brain tumor patients. Additionally, the self-reported nature of the data might introduce response biases, particularly social desirability bias, where patients might underreport their levels of anxiety to align with cultural norms. The study also did not account for potential confounders such as the severity of symptoms, the presence of psychological comorbidities, or the use of coping mechanisms, which could influence death anxiety and QoL scores. Lastly, the cultural context of Saudi Arabia, with its unique social and religious factors, may limit the applicability of these findings to other settings.

## Conclusions

This study highlights the significant psychosocial impact of brain tumors on Saudi Arabian patients, particularly regarding death anxiety and QoL. Our findings emphasize the importance of considering demographic factors such as gender, educational attainment, and marital status in assessing and addressing death anxiety. The inverse relationship between chronic medical conditions and death anxiety, as well as the lower anxiety in advanced tumor stages, underscores the need for further research into resilience and adaptation mechanisms. The positive role of spiritual well-being and meaning in life in enhancing QoL suggests that holistic care approaches are essential for improving patient outcomes. Future research should focus on longitudinal studies to better understand the causal relationships and develop targeted interventions to support the psychosocial well-being of brain tumor patients.
